# Non-Volatile Metabolite Changes in Green Tea Following Helium-Flushed Enclosed-Chamber Pretreatment of Fresh Leaves Across Five Tea Cultivars

**DOI:** 10.3390/foods15203747

**Published:** 2026-10-21

**Authors:** Mi Hu, Yunqi Huang, Niuniu Shi, Wendi Zhang, Yihu Guan, Yapeng Li, Qianwen Sha, Qiongfen He, Qiaomei Wang, Xiujuan Deng, Wenxia Yuan, Run Yang, Huimin E, Xiaohui Wan, Mingshan Zhou, Baijuan Wang, Xinghua Wang

**Affiliations:** 1 College of Tea Science, Yunnan Agricultural University, Kunming 650201, China; 2 College of Continuing Education, Yunnan Agricultural University, Kunming 650201, China

**Keywords:** green tea, helium-flushed enclosed-chamber pretreatment, tea cultivar, non-volatile metabolites

## Abstract

Postharvest atmospheric pretreatment can alter tea-leaf metabolism, yet its influence on the non-volatile composition retained after green tea processing is not well understood. Fresh leaves from five tea cultivars were subjected to helium-flushed enclosed-chamber pretreatment or conventional spreading and were then processed into green tea under the same manufacturing procedure. HPLC and untargeted LC–MS metabolomics were used to examine treatment-associated differences in non-volatile composition. HPLC revealed significant differences in catechins, gallic acid, and caffeine, although the response patterns varied among the five cultivar comparisons. Of 2304 MS/MS-annotated metabolic features detected by LC–MS, 2291 with pooled QC relative standard deviations (RSDs) ≤ 30% were retained for further analysis. Among these, 232, 169, 10, and 30 met the predefined differential screening criteria in Huangshan Quntizhong, Longjing 43, Zhongbai 1, and Zhonghuang 1, respectively, whereas none met all criteria in Jiukengzao. The differential features were mainly annotated as amino acid-related, lipid-related, and shikimate- and phenylpropanoid-related compounds. KEGG mapping associated these features with pathways involving amino acid, lipid, carbohydrate, and secondary metabolism. Overall, helium-flushed enclosed-chamber pretreatment was associated with changes in the non-volatile composition retained in processed green tea, with the magnitude of the detected differences varying among cultivars.

## 1. Introduction

Green tea quality is shaped by both volatile and non-volatile compounds [1]. Non-volatile constituents, including amino acids, catechins, caffeine, phenolic acids, and flavonoids, contribute substantially to tea taste and mouthfeel [2,3], whereas volatile compounds such as alcohols, aldehydes, ketones, esters, and terpenes are important contributors to aroma [1,4]. After harvest, tea leaves remain metabolically active, and postharvest conditions, including temperature, humidity, treatment duration, and atmospheric composition, can influence the transformation of quality-related metabolites [5,6]. Manipulating the postharvest environment of fresh leaves may therefore modify the chemical composition retained in subsequently processed tea.

Oxygen availability is one of the environmental factors associated with postharvest metabolism in fresh tea leaves. Previous studies have reported increased accumulation of γ-aminobutyric acid (GABA) under oxygen-limited or anaerobic treatments, together with changes in glutamic acid and other amino acids [7,8]. Metabolomics studies have also identified broader compositional changes under anaerobic conditions, involving sugars, organic acids, lipids, and phenolic compounds [9]. Oxygen availability may further influence fatty acid-derived metabolism, including reactions associated with the lipoxygenase–hydroperoxide lyase (LOX–HPL) pathway involved in volatile formation [10]. These studies indicate that modified atmospheric conditions during postharvest treatment can affect several metabolic processes in fresh tea leaves and may influence the composition of subsequently processed tea. Anaerobic treatment has also been incorporated as an additional fresh-leaf processing step before green tea manufacture and compared with regular withering, providing a processing-based framework for evaluating modified atmospheric pretreatments [11].

Research on anaerobic or oxygen-limited treatment of fresh tea leaves has mainly focused on GABA enrichment, optimization of treatment conditions, and selected metabolic or flavor-related changes [12,13,14]. The broader effects of such pretreatments on the non-volatile composition retained in processed green tea, however, remain less well characterized. Tea cultivar is an important source of variation in the non-volatile metabolite composition of processed tea [15]. Evaluating several cultivars within the same experimental framework can therefore help characterize the range of treatment-associated compositional differences across different tea materials. LC–MS-based metabolomics, combined with targeted analysis of major quality-related constituents, enables these changes to be examined at both individual-compound and broader metabolic-profile levels.

Five tea cultivars, Jiukengzao (JKZ), Longjing 43 (LJ), Huangshan Quntizhong (HS), Zhongbai 1 (ZB), and Zhonghuang 1 (ZH), were included in this study. Previous studies have used N_2_ or CO_2_ filling and vacuum treatment to establish oxygen-limited or anaerobic conditions for fresh tea leaves [7,11,13]. In the present study, helium was used as a chemically inert flushing gas to displace chamber air during the enclosed-chamber pretreatment rather than as a gas expected to exert a helium-specific biochemical effect. N_2_ can likewise serve as an inert gas for atmospheric displacement, and the present study does not imply that helium is intrinsically superior to N_2_. CO_2_ differs in that elevated CO_2_ can itself affect respiratory and metabolic processes in plant tissues; its use may therefore introduce an additional physiologically active factor. The choice of helium in this study was therefore procedural rather than mechanistic. Because the chamber gas composition was not directly measured and residual O_2_ was not monitored during the 8 h pretreatment, the treatment is described operationally as a helium-flushed enclosed-chamber pretreatment. Conventional fresh-leaf spreading served as the processing reference; accordingly, the experiment compared this additional pretreatment with the conventional green tea processing workflow rather than testing a defined gas composition or oxygen concentration as an isolated factor. After the 8 h pretreatment, treated and control leaves were processed into green tea using the same manufacturing procedure. HPLC and untargeted LC–MS metabolomics were then used to characterize major quality-related constituents and overall non-volatile metabolic profiles in the resulting green teas. The aim was to characterize non-volatile compositional differences associated with fresh-leaf helium-flushed enclosed-chamber pretreatment relative to conventional spreading across five tea cultivars.

## 2. Materials and Methods

### 2.1. Chemicals and Reagents

Helium (purity ≥ 99.999%) was purchased from Qingdao Huayu Gas Co., Ltd. (Qingdao, China). Standards of (+)-catechin (C), gallic acid (GA), gallocatechin (GC), epigallocatechin (EGC), epigallocatechin gallate (EGCG), epicatechin gallate (ECG), epicatechin (EC), gallocatechin gallate (GCG), and caffeine (CAF) were purchased from Shanghai Zhenzhun Biotechnology Co., Ltd. (Shanghai, China). Catechin gallate (CG) was purchased from Shanghai Yuanye Bio-Technology Co., Ltd. (Shanghai, China). Chromatographic-grade acetic acid was obtained from Shanghai Aladdin Biochemical Technology Co., Ltd. (Shanghai, China). Chromatographic-grade methanol and acetonitrile were purchased from Merck (Darmstadt, Germany). Analytical-grade methanol, disodium ethylenediaminetetraacetate (EDTA-2Na), and ascorbic acid were purchased from Sinopharm Chemical Reagent Co., Ltd. (Shanghai, China). Ultrapure water was used throughout the experiments.

### 2.2. Preparation of Tea Samples

Fresh tea leaves from five cultivars were harvested in mid-October 2024 from a tea experimental base in Jufeng Town, Donggang District, Rizhao, China, using a plucking standard of one bud with two to three leaves. The helium-flushed pretreatment groups were designated as Jiukengzao (JKZH), Longjing 43 (LJH), Huangshan Quntizhong (HSH), Zhongbai 1 (ZBH), and Zhonghuang 1 (ZHH), with the corresponding conventional spreading controls designated as JKZ, LJ, HS, ZB, and ZH, respectively. Three independent biological replicates were prepared for each treatment, with 12 kg of fresh leaves used at the start of pretreatment for each replicate. For each cultivar, the helium-flushed pretreatment and conventional spreading control were established using independently collected fresh-leaf samples, with three independent biological replicates included in each treatment.

For each biological replicate, 12 kg of fresh leaves were evenly distributed among five identical 20 L stainless-steel chambers operated simultaneously under the same treatment protocol. The use of five chambers was necessary to accommodate the total leaf mass while maintaining a leaf layer thickness of approximately 3–5 cm. The same helium flow rate, flushing duration, pretreatment duration, preset temperature, relative humidity, and helium-supplement schedule were applied to all five chambers. Thus, the five chambers were treated as subsampling units within one biological replicate rather than as independent experimental replicates. Helium was introduced into each chamber at 2 L/min for 15 min to displace the chamber air. The chambers were then sealed, and the leaves were maintained for 8 h under preset conditions of 30 ± 2 °C and 90 ± 5% relative humidity, with additional helium supplied every 2 h. After pretreatment, leaves from the five chambers were pooled to generate one composite biological replicate. Control leaves were conventionally spread for 8 h without helium exposure under the same nominal temperature and relative humidity conditions. Because O_2_ and CO_2_ concentrations in the chambers were not measured, the experiment was designed to compare the overall helium-flushed enclosed-chamber pretreatment with conventional spreading rather than to test a defined chamber gas composition or oxygen concentration as an isolated factor.

After pretreatment, all samples were processed into green tea using the same manufacturing procedure. Fixation was performed in a 6CST-40 drum-type fixation machine (Zhejiang Chunjiang Tea Machinery Co., Ltd., Hangzhou, China) at 280 ± 10 °C until the leaves reached the appropriate fixation endpoint, which generally required 6–8 min. The endpoint was judged by the following characteristics: the leaf color changed from bright green to dull dark green with loss of surface gloss; the leaves became soft and pliable and could be compressed into a ball without stem breakage; and the grassy odor disappeared with the development of a characteristic tea aroma. The fixed leaves were then rolled for 30 min. The first drying stage was conducted at 100 ± 5 °C until the moisture content reached approximately 15–20%, generally requiring 15–20 min. After cooling and spreading for 30 min, the leaves were dried at 80 ± 5 °C until the final moisture content was below 6%, generally requiring 20–25 min. Moisture content at the intermediate and final drying stages was determined by a gravimetric loss-on-drying method based on the mass difference before and after oven drying. The same processing criteria and equipment settings were applied to all pretreatment and control samples.

### 2.3. HPLC Analysis of Catechins, Gallic Acid, and Caffeine

Catechins, gallic acid, and caffeine were analyzed using a high-performance liquid chromatography (HPLC) system (Agilent 1260 Infinity III, Agilent Technologies, Santa Clara, CA, USA) equipped with a variable wavelength detector (VWD). Chromatographic separation was performed on an Agilent InfinityLab Poroshell 120 EC-C18 column (4.6 mm × 250 mm, 4 μm).

Tea samples were ground to a fine powder under liquid nitrogen. Approximately 0.1000 g of each powdered sample was accurately weighed into a centrifuge tube, and 2.5 mL of preheated 70% (*v*/*v*) aqueous methanol was added. The samples were extracted in an ultrasonic water bath at 70 °C for 1 h. After cooling to room temperature, the extracts were centrifuged at 5000 rpm for 5 min, and the supernatants were collected. The residues were re-extracted with another 2.5 mL of 70% aqueous methanol under the same conditions. The two supernatants were combined and brought to a final volume of 5.0 mL with 70% aqueous methanol. The extracts were then filtered through a 0.22 μm organic-phase membrane filter before HPLC analysis. Catechin stability under the above extraction conditions was not independently assessed in this study. Therefore, possible extraction-related changes in catechin composition during the two 1 h ultrasonic extraction steps at 70 °C cannot be excluded and should be considered when interpreting the HPLC results.

Chromatographic separation was carried out using gradient elution. Mobile phase A consisted of ultrapure water containing 0.1% (*v*/*v*) acetic acid, and mobile phase B consisted of acetonitrile containing 0.1% (*v*/*v*) acetic acid. The gradient program was as follows: 0–5 min, 6% B; 5–20 min, 6–15% B; 20–35 min, 15–25% B; 35–45 min, 25–35% B; 45–50 min, 35–6% B; and 50–55 min, 6% B for column equilibration. The flow rate was 0.9 mL/min, the column temperature was 30 °C, and the injection volume was 5 μL. Detection was performed at 270 nm.

The quantified compounds were gallic acid (GA), (+)-catechin (C), gallocatechin (GC), epigallocatechin (EGC), epigallocatechin gallate (EGCG), epicatechin gallate (ECG), epicatechin (EC), gallocatechin gallate (GCG), catechin gallate (CG), and caffeine (CAF). Individual stock solutions of GA, GC, EGC, C, CAF, EGCG, EC, GCG, CG, and ECG were prepared in chromatographic-grade methanol at 10 mg/mL. Mixed working standard solutions were prepared by dilution with a standard diluent consisting of 10% (*v*/*v*) aqueous acetonitrile containing 5% (*v*/*v*) of a 10 mg/mL EDTA-2Na aqueous solution and 5% (*v*/*v*) of a 10 mg/mL ascorbic acid aqueous solution. For GA, EC, GCG, GC, ECG, and CG, the mixed standard concentrations were 1, 5, 12.5, 50, and 100 μg/mL. For C, EGC, and CAF, the concentrations were 2, 10, 25, 100, and 200 μg/mL. For EGCG, the concentrations were 4, 20, 50, 200, and 400 μg/mL. Calibration curves were constructed by plotting chromatographic peak area against the corresponding standard concentration. Quantification was performed using the external standard method.

### 2.4. LC–MS-Based Untargeted Metabolomics Analysis

Freeze-dried tea samples (25 ± 1 mg) were accurately weighed and extracted with 1000 μL of methanol/acetonitrile/water (2:2:1, *v*/*v*/*v*) containing a mixture of deuterated internal standards, including indole-3-acetic-2,2-d2 acid (2.37585 μmol/L), decanoic acid-d19 (0.156756 μmol/L), glycocholic acid-d4 (0.0851703 μmol/L), and diisobutyl phthalate-3,4,5,6-d4 (3.54145 μmol/L). After vortexing for 30 s, the samples were homogenized at 35 Hz for 4 min and sonicated in an ice-water bath for 5 min. This homogenization–sonication cycle was repeated three times. The samples were then incubated at −40 °C for 1 h. Subsequently, 400 μL of each extract was transferred to a 0.22 μm filter plate and filtered under positive pressure at 6 psi for 120 s. The filtrates were collected for LC–MS analysis. A pooled quality control (QC) sample was prepared by combining equal aliquots of all sample extracts, and four pooled QC samples were included in the analytical sequence. Blank samples were included to monitor potential carryover.

LC–MS analysis was performed using a Vanquish ultra-high-performance liquid chromatography (UHPLC) system coupled to an Orbitrap Exploris 120 high-resolution mass spectrometer (Thermo Fisher Scientific, Waltham, MA, USA). Chromatographic separation was achieved on a Kinetex C18 column (2.1 mm × 50 mm, 2.6 μm; Phenomenex, Torrance, CA, USA). Mobile phase A consisted of water containing 0.01% acetic acid, while mobile phase B consisted of isopropanol/acetonitrile (1:1, *v*/*v*). The gradient program was as follows: 0–0.50 min, 1% B; 0.50–4.00 min, 1–99% B; 4.00–4.50 min, 99% B; 4.50–4.55 min, 99–1% B; and 4.55–6.00 min, 1% B. The flow rate was 0.30 mL/min, the column temperature was 25 °C, the autosampler temperature was 4 °C, and the injection volume was 2 μL.

Mass spectrometric data were acquired in both positive and negative ion modes using Xcalibur software (version 4.4; Thermo Fisher Scientific). MS/MS spectra were acquired using an information-dependent acquisition (IDA) strategy, corresponding to a data-dependent acquisition (DDA)-type workflow, in which precursor ions detected in the full-scan MS survey were selected for subsequent MS/MS acquisition. The sheath and auxiliary gas flow rates were set to 50 and 15 Arb, respectively, and the capillary temperature was maintained at 320 °C. Full-scan MS and MS/MS resolutions were set to 60,000 and 15,000, respectively. Stepped normalized collision energies of 20, 30, and 40 were applied. The spray voltages were set to +3.8 kV in positive ion mode and −3.4 kV in negative ion mode.

Raw LC–MS data were converted to mzXML format using ProteoWizard (version 3.0.24094) and processed using an in-house R workflow based on XCMS (version 3.12.0) for feature detection, extraction, alignment, and peak-area integration. Data preprocessing included RSD-based noise filtering, filtering according to a predefined 50% missing-value criterion, imputation of the remaining missing values using half of the minimum observed value, and normalization using the deuterated internal-standard mixture described above. The LC–MS data were used for relative metabolic profiling based on normalized peak areas rather than for compound-specific absolute quantification. After preprocessing, 35,200 metabolic features were retained, of which 2304 received MS/MS-based annotations. The annotated features were further evaluated using the pooled QC samples, and those with QC RSD > 30% were excluded, resulting in 2291 QC-qualified annotated metabolic features for subsequent analyses. Metabolite annotation was performed using BiotreeDB (version 3.0), which includes reference-standard information, and BT-Plant (version 1.1), a plant-specific metabolite database. Annotations were assigned by matching available MS1 information and MS/MS fragmentation spectra against the reference databases, with retention-time information considered when available. According to the annotation scheme used in the analytical workflow, Level 1 identification required agreement of MS1, MS/MS, and retention time with an authentic standard, whereas Level 2 annotation was based on MS1 and MS/MS matching against reference databases. Because compound-specific identification levels were not available for all retained features, the 2291 retained features are conservatively referred to as QC-qualified annotated metabolic features rather than confirmed metabolites.

### 2.5. Statistical Analysis

HPLC data are presented as mean ± standard deviation (SD) from three independent biological replicates (*n* = 3). For each cultivar, the helium-flushed pretreatment group was compared with the corresponding conventional spreading control using a two-sided Welch’s *t*-test. The resulting *p* values for the ten quantified compounds were adjusted using the Benjamini–Hochberg false discovery rate (FDR) procedure within each cultivar comparison. An FDR-adjusted *p* < 0.05 was considered statistically significant.

Principal component analysis (PCA) of the LC–MS data was performed in R (version 3.5.1) using the stats package (version 3.5.1). Pairwise orthogonal partial least squares discriminant analysis (OPLS–DA) was performed in R (version 3.5.1) using MetaboAnalystR (version 1.0.1) for JKZH vs. JKZ, LJH vs. LJ, HSH vs. HS, ZBH vs. ZB, and ZHH vs. ZH. Model performance was evaluated using *R*^2^X, *R*^2^Y, and *Q*^2^, and 200 permutation tests were performed to assess model stability. Given the limited number of biological replicates (*n* = 3 per group), OPLS–DA was used primarily as an exploratory multivariate analysis and to obtain variable importance in projection (VIP) values.

Differential features were screened separately for each pretreatment–control comparison using a two-sided Welch’s *t*-test followed by Benjamini–Hochberg FDR correction. Features meeting all three predefined criteria—VIP > 1, FDR-adjusted *p* < 0.05, and |log_2_FC| ≥ 1—were considered differential. To reduce confounding by cultivar background, pretreatment and control samples were compared separately within each cultivar. PCA across all samples was used to visualize overall compositional variation, including cultivar-related structure, and was not used to estimate a treatment effect independent of cultivar. Hierarchical clustering heatmaps, Venn diagrams, volcano plots, and differential-feature class/VIP plots were generated using R software (version 4.1.2).

KEGG pathway enrichment analysis was performed separately for each pretreatment–control comparison using the differential features that met the predefined screening criteria. Differential features were mapped to the plant KEGG pathway database, with KEGG-mapped features in the quantitative dataset used as the enrichment background. Pathway enrichment was evaluated using a hypergeometric test, and the resulting *p* values were adjusted using the Benjamini–Hochberg procedure.

## 3. Results

### 3.1. Treatment-Associated Differences in Catechins, Gallic Acid, and Caffeine

HPLC analysis showed variation in the contents of catechins, gallic acid, and caffeine among the green tea samples (Table 1). Among the quantified compounds, EGCG was the most abundant catechin in all samples, with EC and EGC also present at high concentrations.

Pairwise comparisons between the helium-flushed pretreatment group and the corresponding conventional spreading control revealed significant differences in several compounds. In JKZH vs. JKZ, the contents of C, GC, EC, EGC, CG, GCG, ECG, EGCG, and CAF were significantly higher in the pretreatment samples, whereas GA showed no significant difference. In LJH vs. LJ, C and EGC were significantly higher, while GC and GCG were significantly lower; the remaining compounds showed no significant differences.

In HSH vs. HS, GA, C, EC, EGC, ECG, and EGCG were significantly higher in the pretreatment samples, whereas GC, CG, GCG, and CAF did not differ significantly. In ZBH vs. ZB, GA was significantly higher, while C, GC, EC, CG, GCG, and CAF were significantly lower; no significant differences were observed for EGC, ECG, or EGCG. In ZHH vs. ZH, EC, EGC, CG, GCG, ECG, EGCG, and CAF were significantly higher in the pretreatment samples, whereas GA, C, and GC showed no significant differences.

### 3.2. LC–MS Data Quality Assessment and Overall Metabolic Profiles

Untargeted LC–MS metabolomics was used to characterize the non-volatile metabolic profiles of green teas following helium-flushed enclosed-chamber pretreatment or conventional spreading. After data preprocessing, 35,200 metabolic features were retained, of which 2304 received MS/MS-based annotations. Further filtering using a pooled QC RSD threshold of ≤30% retained 2291 QC-qualified annotated metabolic features for subsequent analyses.

**Figure 1:**
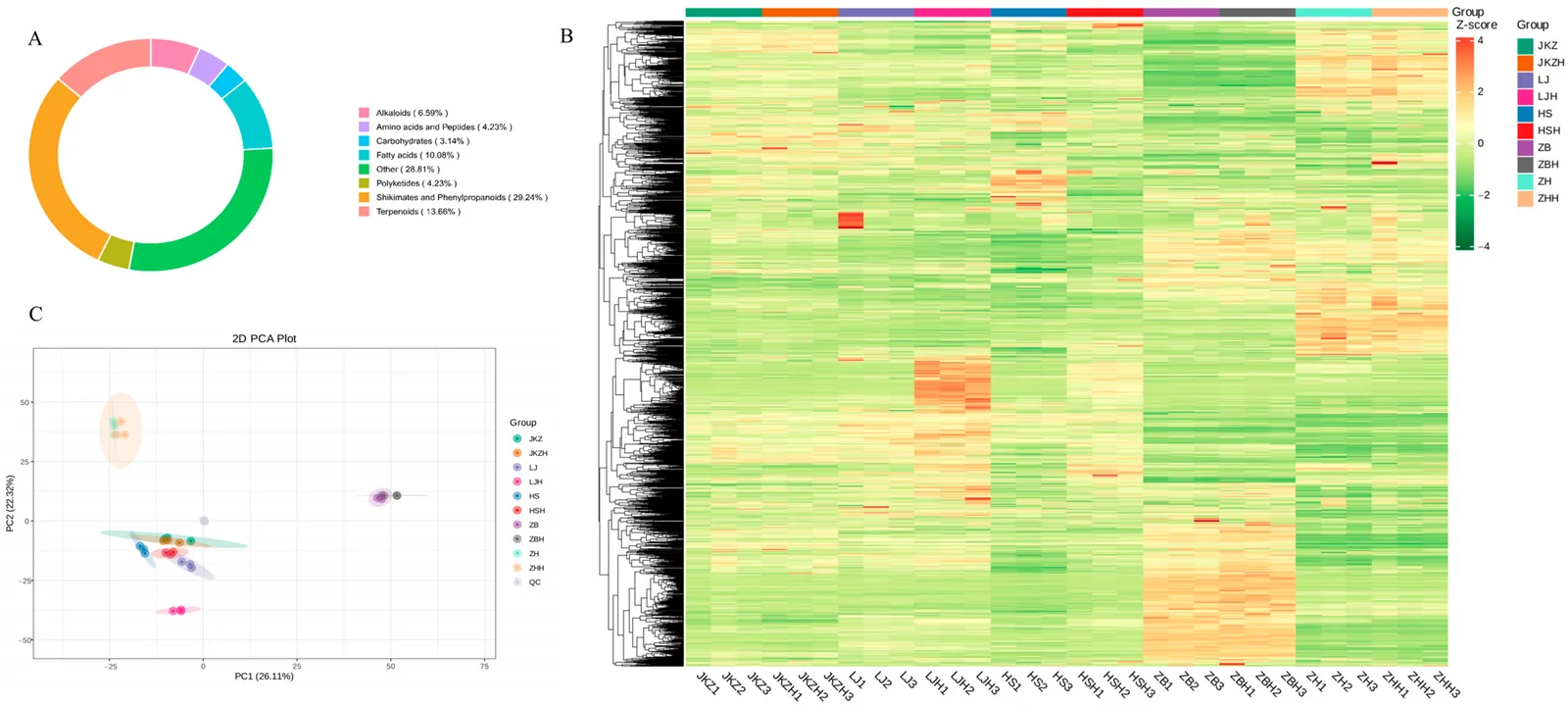
Overall non-volatile metabolic profiles of green teas following helium-flushed enclosed-chamber pretreatment and conventional spreading. (**A**) HMDB classification of 2291 QC-qualified annotated metabolic features. (**B**) Hierarchical clustering heatmap. (**C**) PCA score plot; PC1 and PC2 explained 26.11% and 22.32% of the variance, respectively. H-suffixed groups indicate helium-flushed pretreatment; QC, pooled quality control samples.

HMDB-based chemical classification showed that the 2291 QC-qualified annotated metabolic features were distributed across multiple chemical categories (Figure 1A). Shikimates and phenylpropanoids accounted for the largest proportion (29.24%), followed by other annotated metabolites (28.81%), terpenoids (13.66%), and fatty acids (10.08%). The hierarchical clustering heatmap showed variation in metabolite abundance patterns among the tea samples, with different degrees of separation between pretreatment and corresponding conventional spreading control samples across the five comparisons (Figure 1B).

PCA further illustrated variation in the overall metabolic profiles (Figure 1C). PC1 and PC2 explained 26.11% and 22.32% of the total variance, respectively, accounting for 48.43% of the cumulative variance. ZB and ZH samples showed relatively distinct distributions from several of the other tea samples, while the degree of separation between pretreatment and control samples differed among the five comparisons.

Separate OPLS–DA models were constructed for JKZH vs. JKZ, LJH vs. LJ, HSH vs. HS, ZBH vs. ZB, and ZHH vs. ZH (Figure S1). The five pairwise models showed separation between pretreatment and control samples in the OPLS–DA score space, with Q^2^ values ranging from 0.627 to 0.956. The corresponding results of the 200 permutation tests are shown in Figure S2.

### 3.3. Differentially Abundant Annotated Metabolic Features Associated with Helium-Flushed Enclosed-Chamber Pretreatment

**Figure 2:**
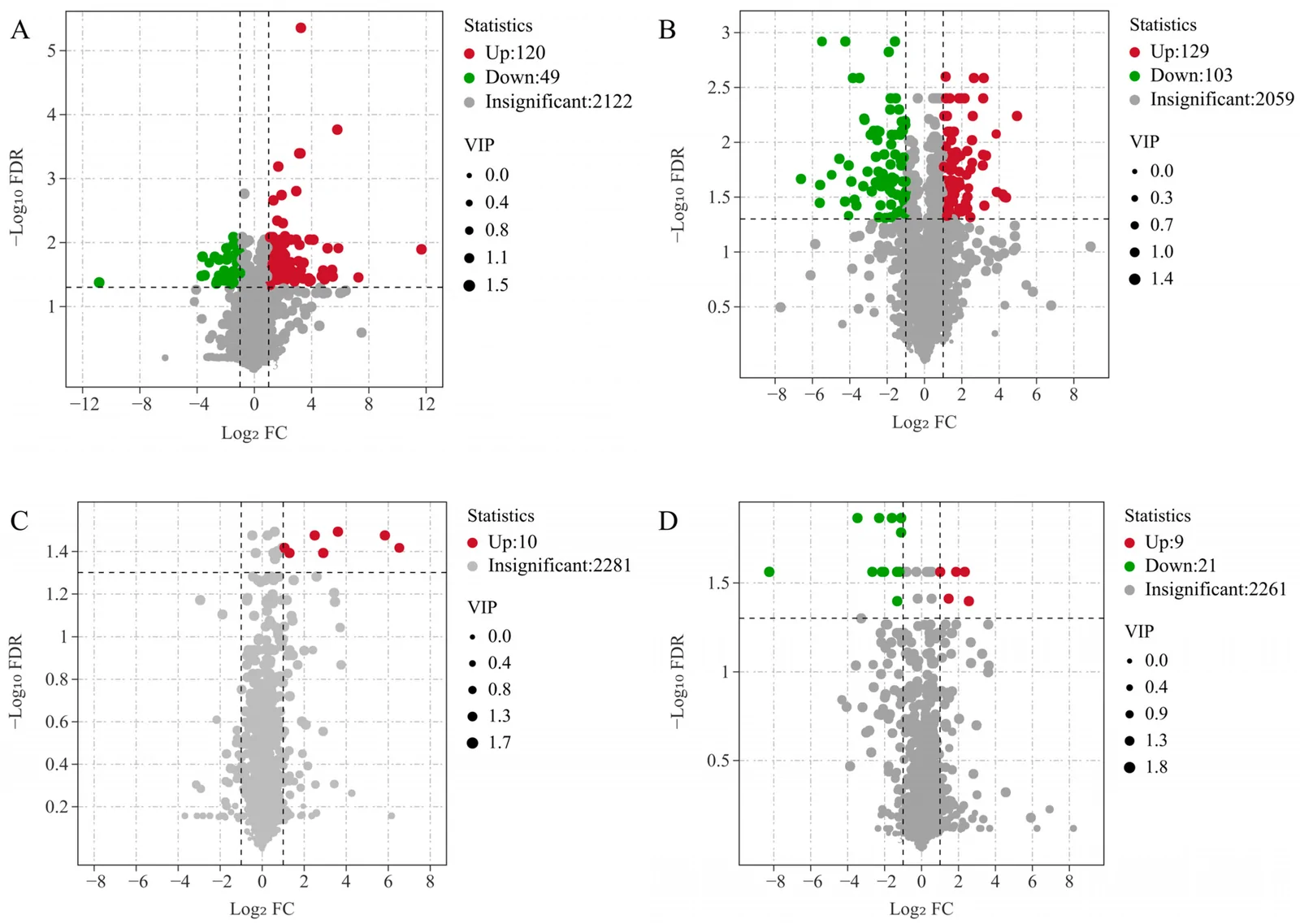
Volcano plots of differential features in four cultivar comparisons. (**A**) LJH vs. LJ; (**B**) HSH vs. HS; (**C**) ZBH vs. ZB; (**D**) ZHH vs. ZH. Differential features were screened using VIP > 1, FDR-adjusted *p* < 0.05, and |log_2_FC| ≥ 1. The vertical dashed lines indicate log_2_FC values of −1 and 1, and the horizontal dashed line indicates an FDR-adjusted *p* value of 0.05.

Differentially abundant annotated metabolic features (hereafter, differential features) were screened from the 2291 QC-qualified MS/MS-annotated metabolic features using the predefined criteria of VIP > 1, FDR-adjusted *p* < 0.05, and |log_2_FC| ≥ 1 (Figure 2A–D). The complete list of differential features is provided in Table S2. HSH vs. HS contained the largest number of differential features (232), including 129 with higher abundance and 103 with lower abundance in the pretreatment samples. LJH vs. LJ contained 169 differential features, including 120 with higher abundance and 49 with lower abundance. ZBH vs. ZB contained 10 differential features, all showing higher abundance in the pretreatment samples. ZHH vs. ZH contained 30 differential features, including 9 with higher abundance and 21 with lower abundance. No features in JKZH vs. JKZ met all predefined screening criteria.

**Figure 3:**
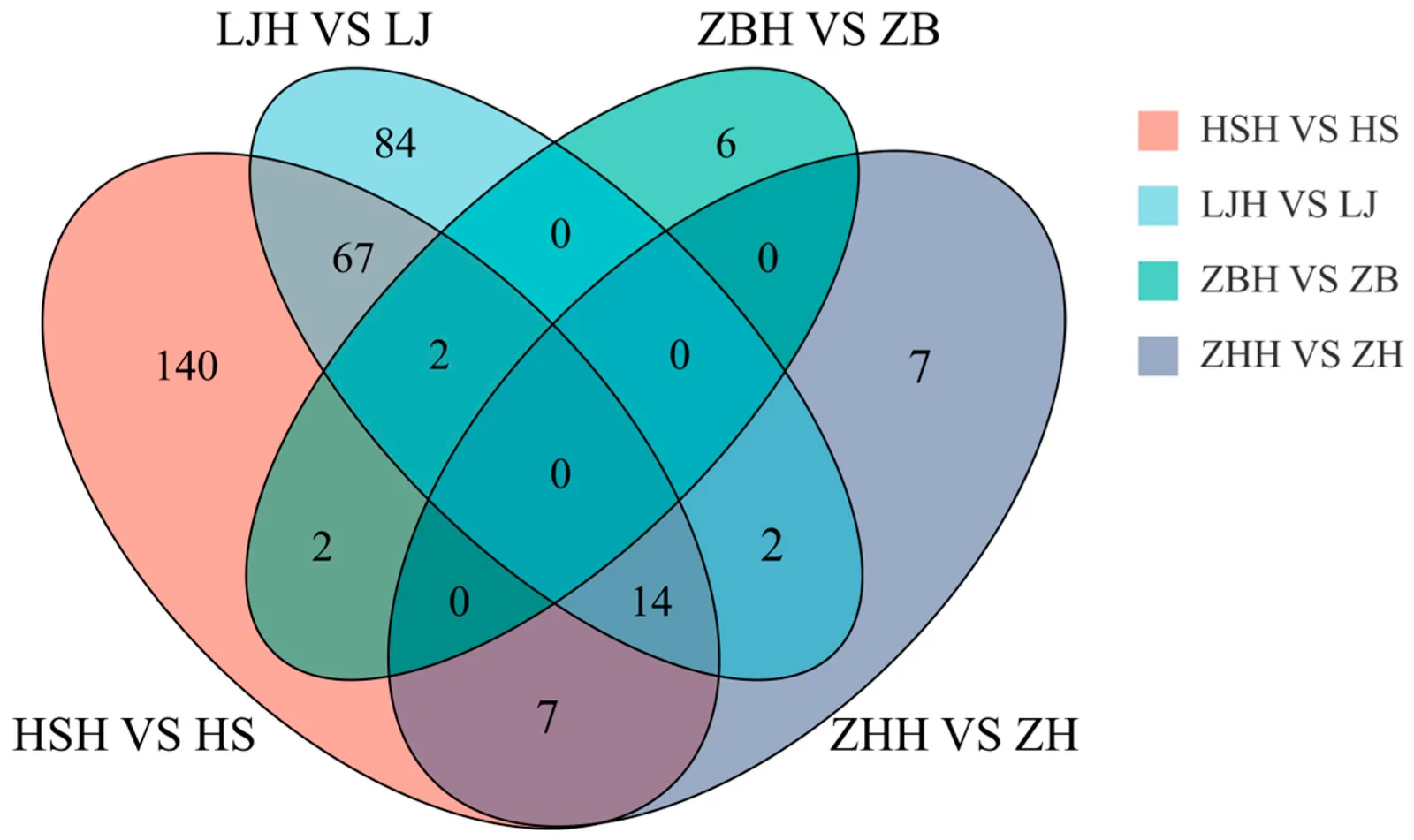
Venn diagram of differential features among four cultivar comparisons. Numbers indicate shared and comparison-specific differential features identified in HSH vs. HS, LJH vs. LJ, ZBH vs. ZB, and ZHH vs. ZH.

Venn analysis of the four comparisons containing differential features identified 331 unique differential features (Figure 3). Of these, 140, 84, 6, and 7 were specific to HSH vs. HS, LJH vs. LJ, ZBH vs. ZB, and ZHH vs. ZH, respectively. A total of 67 differential features were shared exclusively between HSH vs. HS and LJH vs. LJ. Fourteen features were shared among HSH vs. HS, LJH vs. LJ, and ZHH vs. ZH, while two were shared among HSH vs. HS, LJH vs. LJ, and ZBH vs. ZB. No differential features were shared across all four comparisons.

**Figure 4:**
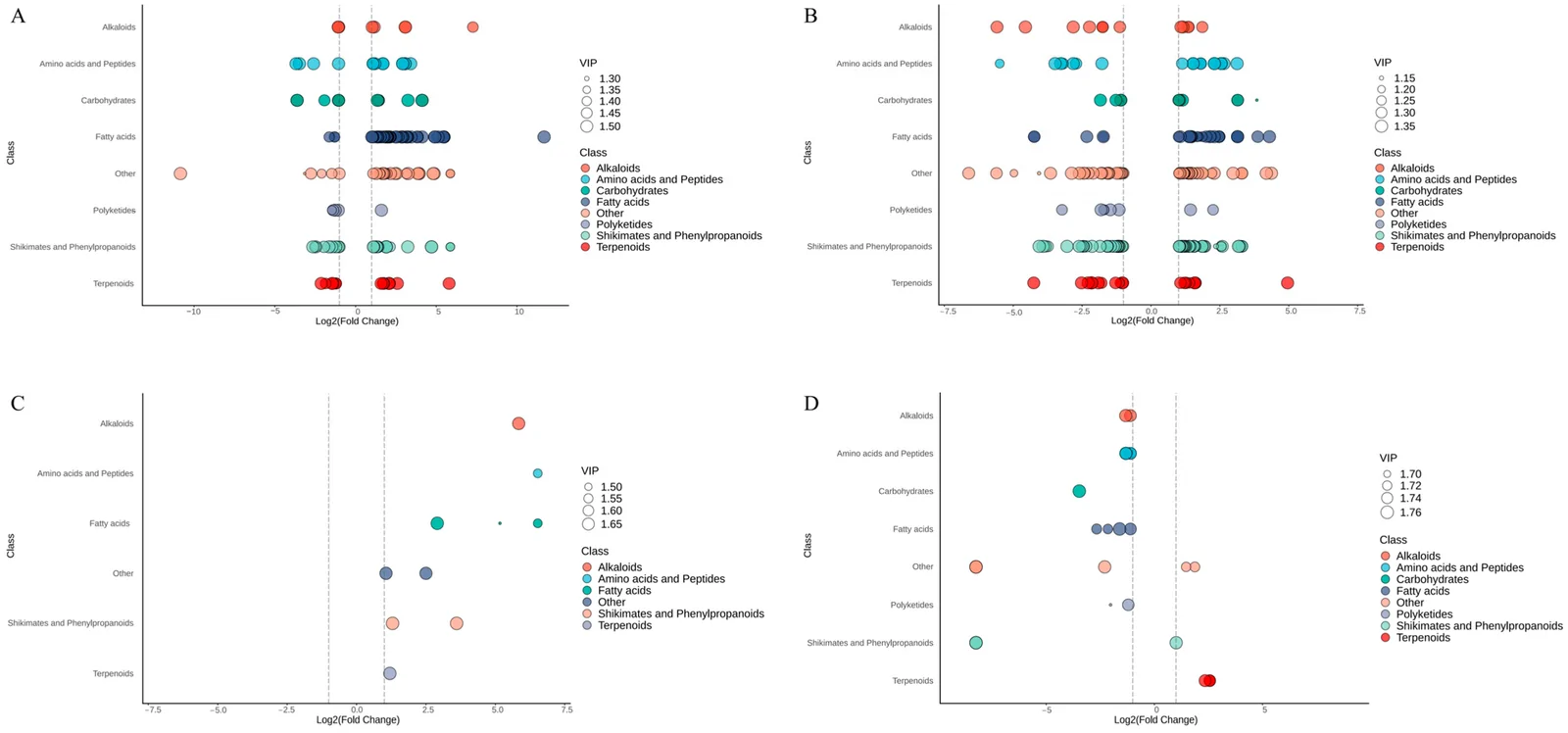
Distribution of differential features across chemical classes. (**A**) LJH vs. LJ; (**B**) HSH vs. HS; (**C**) ZBH vs. ZB; (**D**) ZHH vs. ZH. The vertical dashed lines indicate log_2_FC values of −1 and 1.

Representative differential features were further grouped according to their chemical classes and abundance patterns (Figure 4A–D). These included amino acid- and peptide-related compounds, fatty acid- and lysophospholipid-related compounds, and shikimate- and phenylpropanoid-related compounds. Amino acid-associated metabolites included alanine-, beta-alanine-, and tyrosine-related compounds. Several lysophospholipid-related metabolites were identified in LJH vs. LJ, together with fatty acid-related metabolites. Shikimate- and phenylpropanoid-related metabolites were also represented across the comparisons.

### 3.4. KEGG Pathway Mapping and Enrichment of Differential Features

**Figure 5:**
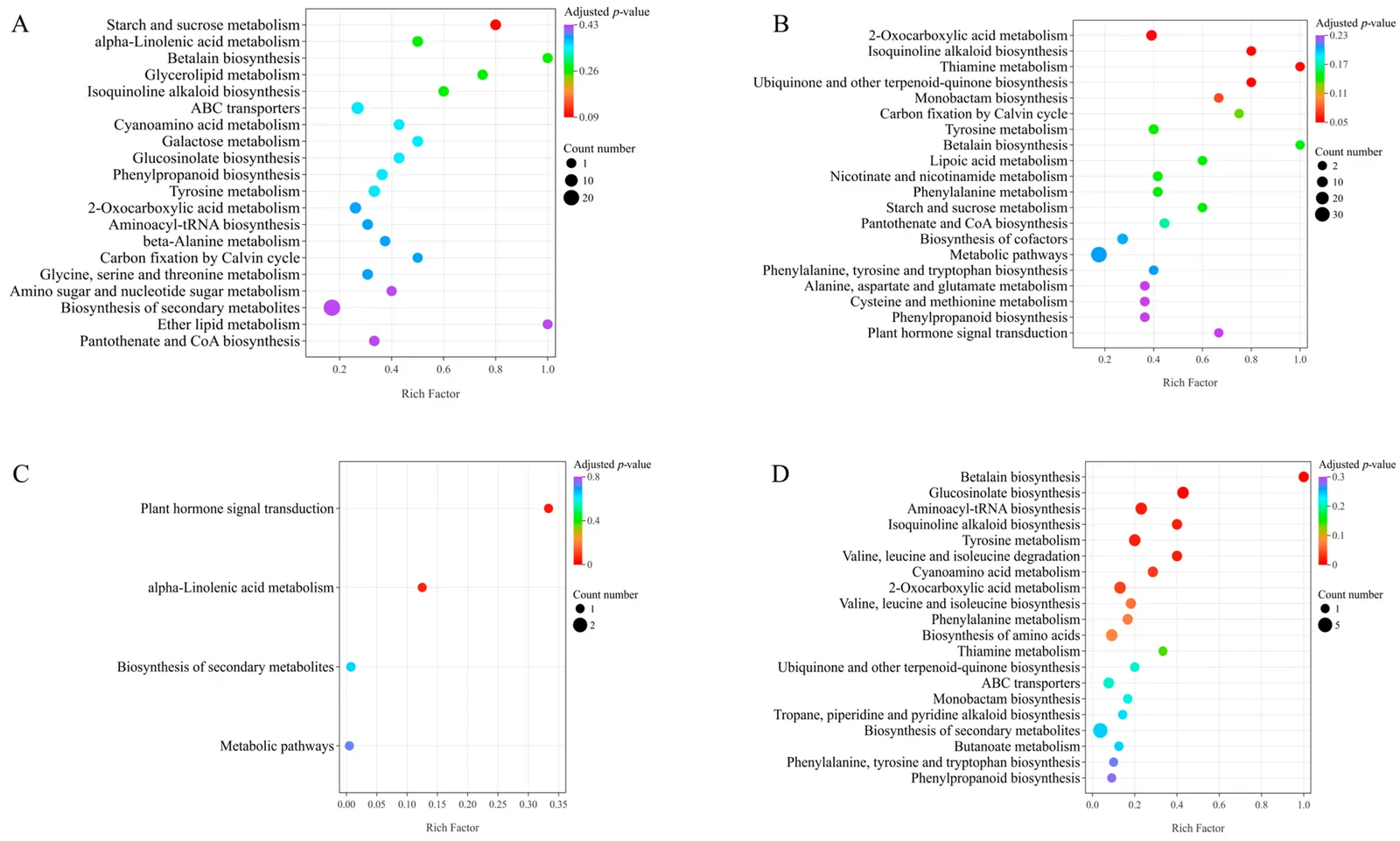
KEGG pathway enrichment analysis of differential features in four cultivar comparisons. (**A**) LJH vs. LJ; (**B**) HSH vs. HS; (**C**) ZBH vs. ZB; (**D**) ZHH vs. ZH. Point size indicates the number of mapped differential features, color represents the BH-adjusted *p* value, and the rich factor reflects the degree of pathway enrichment.

KEGG pathway enrichment analysis was performed separately for the differential features identified in each pretreatment–control comparison (Figure 5A–D). No pathway analysis was conducted for JKZH vs. JKZ because no features met all predefined differential screening criteria.

In LJH vs. LJ (Figure 5A), differential features were mapped to pathways including starch and sucrose metabolism, alpha-linolenic acid metabolism, glycerolipid metabolism, phenylpropanoid biosynthesis, tyrosine metabolism, 2-oxocarboxylic acid metabolism, beta-alanine metabolism, glycine, serine and threonine metabolism, aminoacyl-tRNA biosynthesis, and biosynthesis of secondary metabolites. Among the displayed pathways, starch and sucrose metabolism had the lowest adjusted *p* value, followed by alpha-linolenic acid metabolism and glycerolipid metabolism. None of the displayed pathways reached an adjusted *p* value below 0.05.

In HSH vs. HS (Figure 5B), the mapped pathways included 2-oxocarboxylic acid metabolism, isoquinoline alkaloid biosynthesis, thiamine metabolism, ubiquinone and other terpenoid-quinone biosynthesis, tyrosine metabolism, phenylalanine metabolism, starch and sucrose metabolism, alanine, aspartate and glutamate metabolism, cysteine and methionine metabolism, phenylpropanoid biosynthesis, and plant hormone signal transduction. Relatively low adjusted *p* values were observed for 2-oxocarboxylic acid metabolism, isoquinoline alkaloid biosynthesis, thiamine metabolism, and ubiquinone and other terpenoid-quinone biosynthesis, while the remaining pathways showed higher adjusted *p* values.

In ZBH vs. ZB (Figure 5C), differential features were mapped to four pathways: plant hormone signal transduction, alpha-linolenic acid metabolism, biosynthesis of secondary metabolites, and metabolic pathways. Plant hormone signal transduction and alpha-linolenic acid metabolism had the lowest adjusted *p* values among these pathways, whereas biosynthesis of secondary metabolites and metabolic pathways had higher adjusted *p* values.

In ZHH vs. ZH (Figure 5D), differential features were mapped to pathways including betalain biosynthesis, glucosinolate biosynthesis, aminoacyl-tRNA biosynthesis, isoquinoline alkaloid biosynthesis, tyrosine metabolism, valine, leucine and isoleucine degradation and biosynthesis, cyanoamino acid metabolism, 2-oxocarboxylic acid metabolism, phenylalanine metabolism, biosynthesis of amino acids, phenylpropanoid biosynthesis, and biosynthesis of secondary metabolites. Betalain biosynthesis, glucosinolate biosynthesis, aminoacyl-tRNA biosynthesis, tyrosine metabolism, and several amino acid-related pathways showed relatively low adjusted *p* values among the displayed pathways.

Overall, the KEGG mapping patterns differed among the four pretreatment–control comparisons. LJH vs. LJ and HSH vs. HS contained larger numbers of mapped pathways, whereas only four pathways were represented in ZBH vs. ZB. Amino acid-, lipid-, and secondary metabolism-related pathways were represented in more than one comparison, although their adjusted *p* values and the number of mapped differential features varied among the comparisons.

## 4. Discussion

Fresh-leaf helium-flushed enclosed-chamber pretreatment was associated with changes in the non-volatile composition of the resulting green teas, although the extent and direction of these differences varied among the five cultivar comparisons. HPLC detected treatment-associated differences in catechins, gallic acid, and caffeine, whereas untargeted metabolomics revealed broader metabolic differences in HS and LJ, fewer differential features in ZB and ZH, and no features in JKZ meeting all predefined screening criteria. The absence of screened differential features in JKZ does not conflict with the HPLC results. HPLC quantified selected compounds using authentic standards and detected statistically significant but relatively modest changes in nine compounds. In contrast, the untargeted LC–MS workflow classified a feature as differential only when VIP > 1, FDR-adjusted *p* < 0.05, and |log_2_FC| ≥ 1 were all satisfied. For the nine HPLC-significant compounds in JKZH vs. JKZ, the observed concentration ratios were only approximately 1.06–1.24-fold, which is well below the twofold-change threshold corresponding to |log_2_FC| ≥ 1. Thus, compounds showing statistically significant differences by targeted HPLC would not necessarily meet the more stringent combined criteria used for LC–MS differential-feature screening. Tea cultivars differ in their basal composition and accumulation of amino acids, phenolic acids, flavonoids, and other non-volatile metabolites [16,17,18,19,20,21], which may partly contribute to the different treatment-associated patterns observed here. Cultivar background and subsequent processing steps can also influence the metabolite composition of processed tea [22,23,24]. Accordingly, treatment-associated changes were defined only from pretreatment–control comparisons within the same cultivar. Differences among cultivars in the number, identity, or direction of screened differential features were interpreted as heterogeneity among the five cultivar comparisons rather than as direct evidence of a cultivar × pretreatment interaction.

Previous studies using N_2_, CO_2_, vacuum, or other anaerobic treatments have reported changes in GABA and other amino acid-related metabolites, as well as changes involving carbohydrates, lipids, and phenolic compounds in fresh tea leaves [7,8,13,25]. The present study showed partial overlap with these reported responses, particularly in the amino acid-related and lipid-related annotations detected in several cultivar comparisons. However, direct comparison among these treatments should be made cautiously because the studies differed in tea material, treatment atmosphere, treatment duration, pressure conditions, sampling stage, and analytical approach. In addition, many previous studies characterized fresh leaves after atmospheric treatment, whereas the present study analyzed the non-volatile composition retained in finished green tea after fixation, rolling, and drying. Because no parallel N_2_-, CO_2_-, vacuum-, or chamber-matched air-flushing treatments were included in the present experiment, the current design does not allow any of the detected metabolic changes to be classified as helium-specific. The observed differences are therefore interpreted as compositional changes associated with the overall helium-flushed enclosed-chamber pretreatment.

Amino acid-related compounds constituted an important part of the differential annotations, particularly in HS and LJ. Corresponding KEGG mappings included beta-alanine metabolism, glycine, serine and threonine metabolism, tyrosine metabolism, alanine, aspartate and glutamate metabolism, and 2-oxocarboxylic acid metabolism. None of the displayed pathways in HS or LJ reached an adjusted *p* value below 0.05; these mappings therefore provide biochemical context rather than evidence of coordinated pathway-level regulation. Previous studies have reported changes in GABA and other amino acid-related metabolites following anaerobic or vacuum treatment of fresh tea leaves [25,26], while nitrogen treatment has been associated with differences in GABA accumulation depending on treatment temperature and duration [27]. These studies support the sensitivity of amino acid composition to modified postharvest atmospheres, although their underlying mechanisms cannot be assumed to apply directly to the helium-flushed enclosed-chamber pretreatment used here.

Lipid- and phenylpropanoid-related compounds also contributed to the observed differences. Several lysophospholipid- and fatty acid-related metabolites were identified in LJ, where alpha-linolenic acid metabolism and glycerolipid metabolism were among the mapped pathways with relatively low adjusted *p* values. Phenylpropanoid-related annotations were represented across several comparisons. Previous lipidomic and metabolomic studies have shown changes in membrane lipids, fatty acids, and phenylpropanoid-related compounds during green tea manufacture, particularly during fixation [28,29], and fixation conditions can also influence amino acids, flavonoids, and other non-volatile constituents [30,31]. Because the present measurements were made in finished green tea, the detected differences represent compositional outcomes retained after fixation, rolling, and drying rather than changes measured immediately after pretreatment. The KEGG results should also be interpreted at the pathway-context level. HS and LJ contained the largest numbers of differential features but showed no pathways with adjusted *p* < 0.05, whereas the smaller differential-feature sets in ZB and ZH mapped to several pathways with relatively low adjusted *p* values. Because individual metabolites can map to multiple KEGG categories, enrichment outcomes depend on the input metabolite set, enrichment background, and pathway database [32]. Thus, the KEGG results describe pathway-level associations rather than pathway activation or inhibition.

The present study compared the overall helium-flushed enclosed-chamber pretreatment with conventional spreading rather than a defined chamber gas composition or oxygen condition. Helium was used to displace chamber air, while the pretreatment samples were enclosed in chambers and the controls were conventionally spread. The five chambers within each biological replicate were operated simultaneously using the same chamber type and treatment protocol. However, O_2_ and CO_2_ concentrations were not directly measured, and the gas composition of individual chambers was not monitored. Thus, possible chamber-to-chamber variation in the actual atmosphere could not be assessed. The contributions of oxygen availability, chamber atmosphere, gas exchange, CO_2_ accumulation, and water loss therefore cannot be separated within the present design. The observed differences are best interpreted as compositional changes associated with the overall helium-flushed enclosed-chamber pretreatment. Further work using direct gas monitoring, chamber-matched controls, and sequential sampling could help determine when these differences arise and how they are retained during subsequent green tea processing. In addition, catechin stability under the HPLC extraction conditions was not independently assessed, and possible extraction-related changes during the two successive 1 h ultrasonic extractions at 70 °C cannot be excluded when interpreting the HPLC results.

## 5. Conclusions

Helium-flushed enclosed-chamber pretreatment was associated with changes in the non-volatile composition retained in processed green tea, although the magnitude and pattern of these changes differed among the five cultivar comparisons. HPLC showed treatment-associated differences in catechins, gallic acid, and caffeine. Untargeted LC–MS further showed broader metabolic differences in HS and LJ, fewer differential features in ZB and ZH, and no features in JKZ that met all predefined screening criteria. Most differential features were related to amino acids, lipids, and shikimate- and phenylpropanoid-derived compounds. These changes involved several compounds associated with green tea quality, indicating that the pretreatment may modulate the quality-related chemical composition of the final product. However, because the direction of these changes differed among cultivars, and sensory properties, antioxidant capacity, and other functional quality attributes were not directly evaluated, the present results do not demonstrate an overall improvement in green tea quality. Because chamber O_2_ and CO_2_ concentrations were not measured directly, reduced oxygen availability was inferred from the flushing procedure rather than experimentally confirmed. The observed differences should therefore be interpreted as responses to the overall helium-flushed enclosed-chamber pretreatment rather than to a defined oxygen condition or to helium itself.

## Figures and Tables

**Table 1:** Effects of helium-flushed enclosed-chamber pretreatment of fresh tea leaves on the contents of gallic acid, catechins, and caffeine in green tea from five tea cultivars.

Compound (mg/g)	JKZ	JKZH	LJ	LJH	HS	HSH	ZB	ZBH	ZH	ZHH
GA	0.33 ± 0.01	0.34 ± 0.01	0.70 ± 0.02	0.69 ± 0.02	0.34 ± 0.01	0.44 ± 0.01 **	0.58 ± 0.01	0.62 ± 0.01 *	0.31 ± 0.00	0.32 ± 0.01
C	0.44 ± 0.00	0.54 ± 0.00 ***	0.51 ± 0.01	0.62 ± 0.02 **	0.31 ± 0.01	0.57 ± 0.01 ***	0.10 ± 0.00	0.06 ± 0.00 ***	0.64 ± 0.01	0.63 ± 0.02
GC	2.16 ± 0.06	2.67 ± 0.08 **	4.35 ± 0.10	2.82 ± 0.07 ***	2.34 ± 0.03	2.28 ± 0.03	1.28 ± 0.02	1.22 ± 0.02 *	5.42 ± 0.15	5.71 ± 0.13
EC	53.19 ± 0.81	61.35 ± 0.93 **	40.96 ± 0.77	40.64 ± 0.19	37.83 ± 0.07	49.87 ± 0.87 **	37.57 ± 0.15	32.92 ± 0.61 *	60.55 ± 0.64	68.22 ± 0.15 **
EGC	35.42 ± 0.58	42.24 ± 0.51 ***	27.18 ± 0.61	31.45 ± 0.50 **	33.16 ± 0.54	37.75 ± 0.96 **	35.56 ± 0.67	34.21 ± 0.33	41.18 ± 0.98	47.08 ± 0.15 *
CG	2.56 ± 0.04	2.92 ± 0.04 **	2.78 ± 0.07	2.91 ± 0.02	2.88 ± 0.05	2.95 ± 0.07	4.30 ± 0.05	4.13 ± 0.02 *	2.34 ± 0.06	2.90 ± 0.04 ***
GCG	1.22 ± 0.03	1.29 ± 0.01 *	1.52 ± 0.01	1.49 ± 0.01 *	1.46 ± 0.01	1.42 ± 0.04	1.91 ± 0.04	1.73 ± 0.03 *	1.19 ± 0.01	1.46 ± 0.01 ***
ECG	21.09 ± 0.35	24.56 ± 0.17 **	18.37 ± 0.15	18.06 ± 0.12	14.28 ± 0.08	18.16 ± 0.45 **	26.25 ± 0.42	26.40 ± 0.15	19.26 ± 0.19	23.75 ± 0.12 ***
EGCG	85.41 ± 1.47	100.32 ± 0.54 **	76.12 ± 1.91	79.98 ± 0.94	70.30 ± 1.50	82.62 ± 2.27 **	110.31 ± 1.80	108.12 ± 0.11	71.76 ± 0.76	88.74 ± 0.54 ***
CAF	28.83 ± 0.46	33.65 ± 0.13 **	29.65 ± 0.25	29.28 ± 0.14	26.09 ± 0.06	26.42 ± 0.42	49.14 ± 0.12	47.56 ± 0.17 **	29.25 ± 0.35	36.58 ± 0.05 **

**Note:** Data are presented as mean ± SD from three independent biological replicates (*n* = 3). Asterisks indicate significant differences between pretreatment and the corresponding control within each cultivar based on FDR-adjusted *p* values (* *q* < 0.05, ** *q* < 0.01, *** *q* < 0.001). GA, gallic acid; C, (+)-catechin; GC, gallocatechin; EC, epicatechin; EGC, epigallocatechin; CG, catechin gallate; GCG, gallocatechin gallate; ECG, epicatechin gallate; EGCG, epigallocatechin gallate; CAF, caffeine.

## Data Availability

The original contributions presented in this study are included in the article and Supplementary Materials. Further inquiries can be directed to the corresponding authors.

## Supplementary Materials

The following supporting information can be downloaded at: https://www.mdpi.com/article/10.3390/foods15203747/s1, Figure S1: OPLS–DA score plots of green tea samples in the five pretreatment–control comparisons. (A) JKZH vs. JKZ; (B) LJH vs. LJ; (C) HSH vs. HS; (D) ZBH vs. ZB; (E) ZHH vs. ZH; Figure S2: Permutation tests of the five pairwise OPLS–DA models based on 200 permutations. (A) JKZH vs. JKZ; (B) LJH vs. LJ; (C) HSH vs. HS; (D) ZBH vs. ZB; (E) ZHH vs. ZH; Figure S3: Representative HPLC chromatograms of the mixed reference standard and green tea samples from the five cultivar comparisons. (A) Mixed reference standard; (B) JKZH; (C) JKZ; (D) ZHH; (E) ZH; (F) ZBH; (G) ZB; (H) HSH; (I) HS; (J) LJH; and (K) LJ. JKZ, ZH, ZB, HS, and LJ represent conventional-spreading controls, whereas JKZH, ZHH, ZBH, HSH, and LJH represent the corresponding helium-flushed enclosed-chamber pretreatment groups; Table S1: Annotation and QC information for the 2291 QC-qualified annotated metabolic features used in subsequent LC–MS analyses; Table S2: Differential features identified in the five cultivar comparisons.
