# Supplementary material for: Non-Volatile Metabolite Changes in Green Tea Following Helium-Flushed Enclosed-Chamber Pretreatment of Fresh Leaves Across Five Tea Cultivars
Source: Foods. 2026 Oct 21;15(20):3747. doi: 10.3390/foods15203747 (PMC13704259; doi:10.3390/foods15203747)
Supplement: Supplementary file 1 [file foods-15-03747-s001.zip › Figure and Table Caption Notes.pdf]

Figure S1: OPLS–DA score plots of green tea samples in the five pretreatment–control comparisons. (A) JKZH vs. JKZ; (B) LJH vs. LJ; (C) HSH vs. HS; (D) ZBH vs. ZB; (E) ZHH vs. ZH;

Figure S2: Permutation tests of the five pairwise OPLS–DA models based on 200 permutations. (A) JKZH vs. JKZ; (B) LJH vs. LJ; (C) HSH vs. HS; (D) ZBH vs. ZB; (E) ZHH vs. ZH;

Figure S3: Representative HPLC chromatograms of the mixed reference standard and green tea samples from the five cultivar comparisons. (A) Mixed reference standard; (B) JKZH; (C) JKZ; (D) ZHH; (E) ZH; (F) ZBH; (G) ZB; (H) HSH; (I) HS; (J) LJH; and (K) LJ. JKZ, ZH, ZB, HS, and LJ represent conventional-spreading controls, whereas JKZH, ZHH, ZBH, HSH, and LJH represent the corresponding helium-flushed enclosed-chamber pretreatment groups;

Table S1: Annotation and QC information for the 2291 QC-qualified annotated metabolic features used in subsequent LC–MS analyses;

Table S2: Differential features identified in the five cultivar comparisons.
