# Supplementary material for: Non-Volatile Metabolite Changes in Green Tea Following Helium-Flushed Enclosed-Chamber Pretreatment of Fresh Leaves Across Five Tea Cultivars
Source: Foods. 2026 Oct 21;15(20):3747. doi: 10.3390/foods15203747 (PMC13704259; doi:10.3390/foods15203747)
Supplement: Supplementary file 1 [file foods-15-03747-s001.zip › Figure S3. Representative HPLC chromatograms of the mixed reference standard and green tea samples from the five cultivar comparisons.pdf]

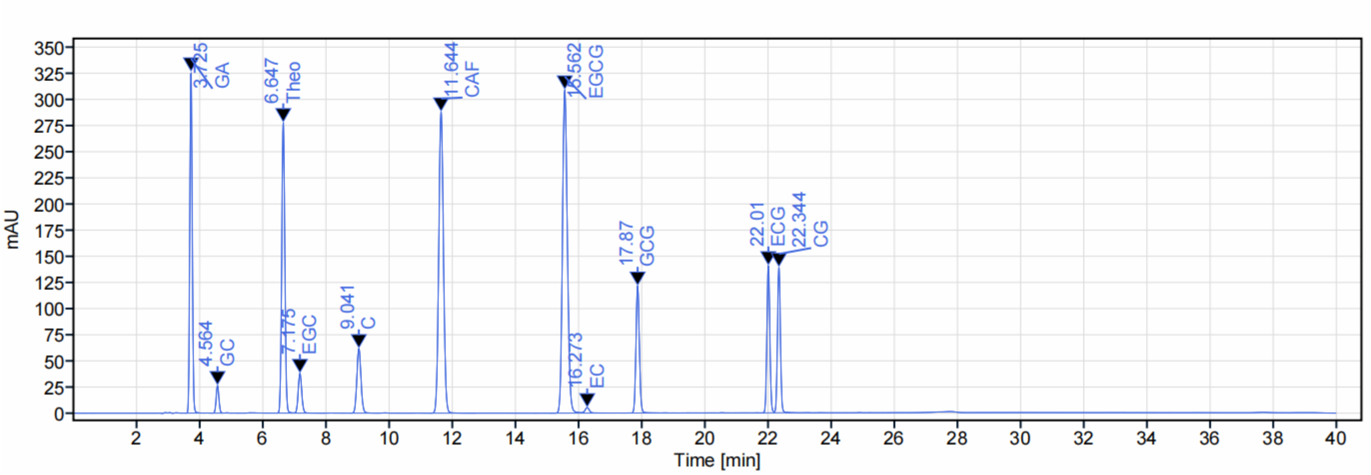

(A) Mixed reference standard

Figure S3. Representative HPLC chromatograms of the mixed reference standard and green tea samples from the five cultivar comparisons. (A) Mixed reference standard; (B) JKZH; (C) JKZ; (D) ZHH; (E) ZH; (F) ZBH; (G) ZB; (H) HSH; (I) HS; (J) LJH; and (K) LJ. JKZ, ZH, ZB, HS, and LJ represent conventional-spreading controls, whereas JKZH, ZHH, ZBH, HSH, and LJH represent the corresponding helium-flushed enclosed-chamber pretreatment groups.

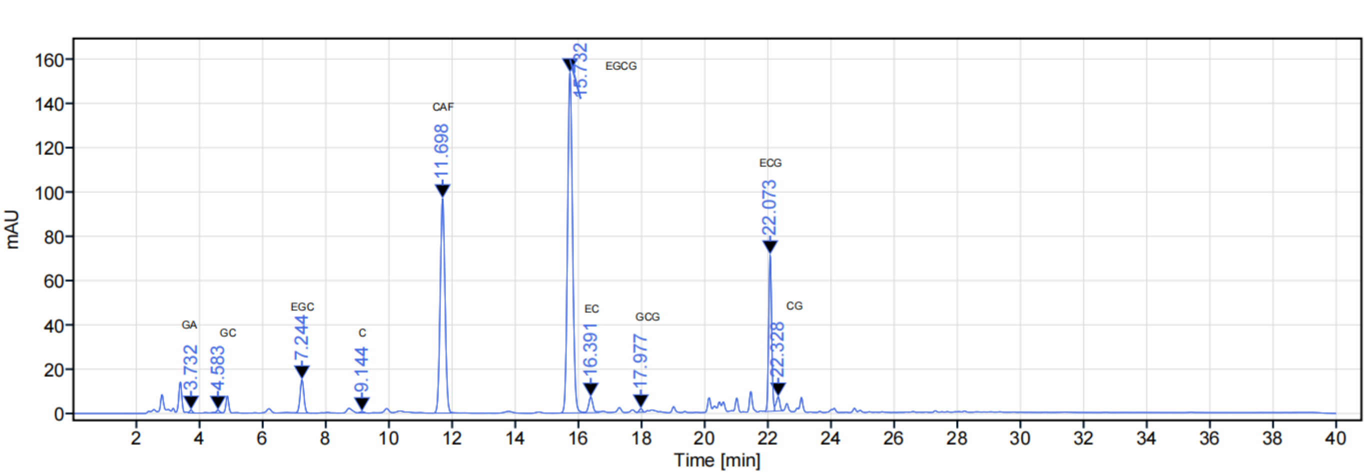

mAU
160
140
120
100
80
60
40
20
0
2
4
6
8
10
12
14
16
18
20
22
24
26
28
30
32
34
36
38
40
Time [min]
GA
3.732
GC
4.583
EGC
7.244
C
9.144
CAF
11.698
15.732
EGCG
EC
16.391
GCG
17.977
ECG
22.073
CG
22.328

(B) JKZH

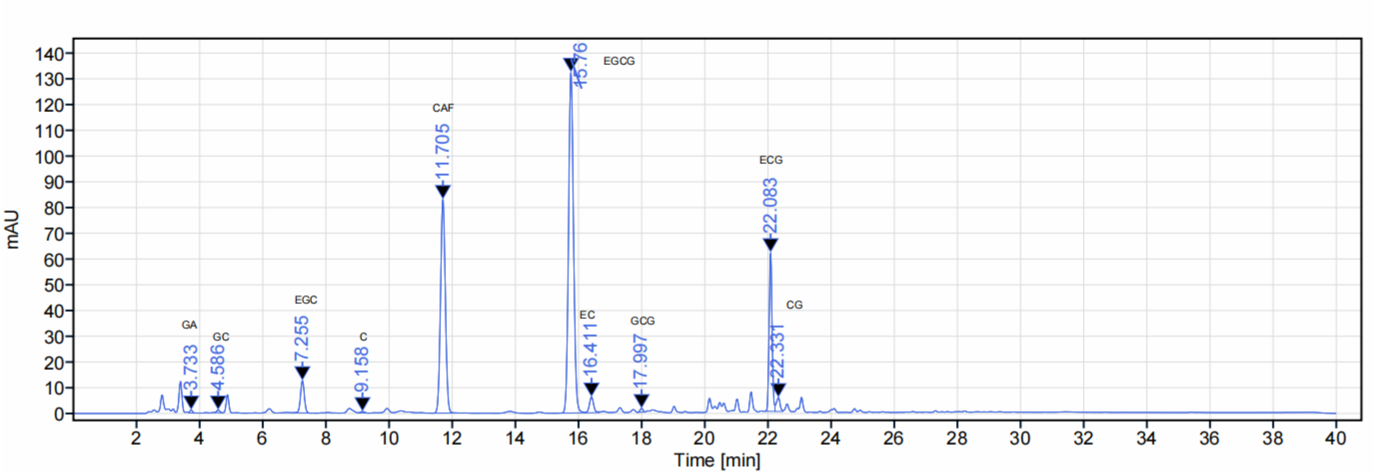
mAU
140
130
120
110
100
90
80
70
60
50
40
30
20
10
0
2
4
6
8
10
12
14
16
18
20
22
24
26
28
30
32
34
36
38
40
Time [min]
GA
3.733
GC
4.586
EGC
7.255
C
9.158
CAF
11.705
15.76
EGCG
EC
16.411
GCG
17.997
ECG
22.083
CG
22.331

(C) JKZ

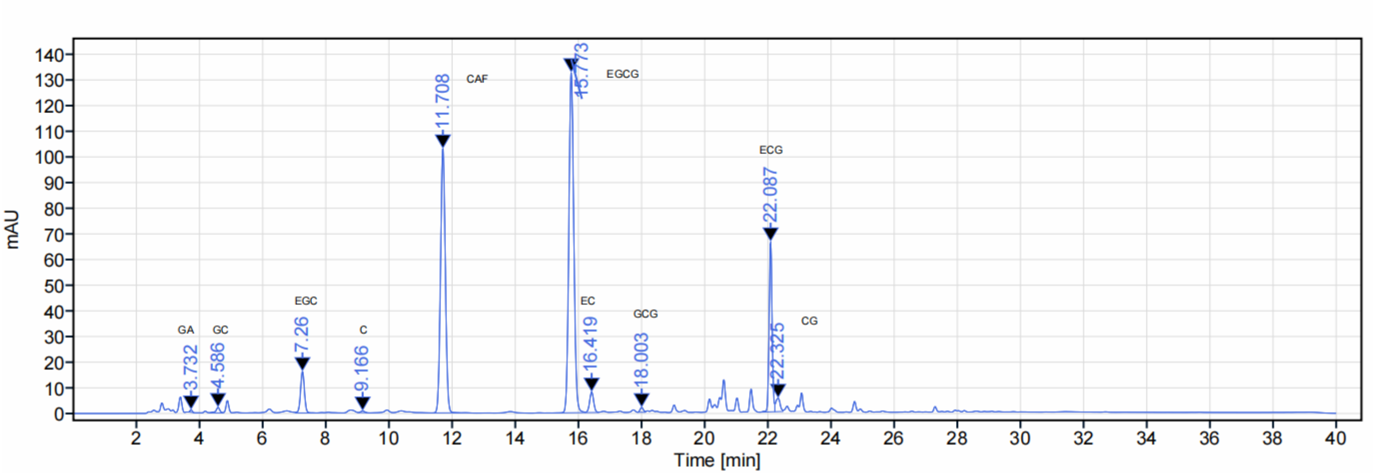
mAU
140
130
120
110
100
90
80
70
60
50
40
30
20
10
0
GA
GC
EGC
C
CAF
EGCG
EC
GCG
ECG
CG
3.732
4.586
7.26
9.166
11.708
15.773
16.419
18.003
22.087
22.325
2
4
6
8
10
12
14
16
18
20
22
24
26
28
30
32
34
36
38
40
Time [min]

(D) ZHH

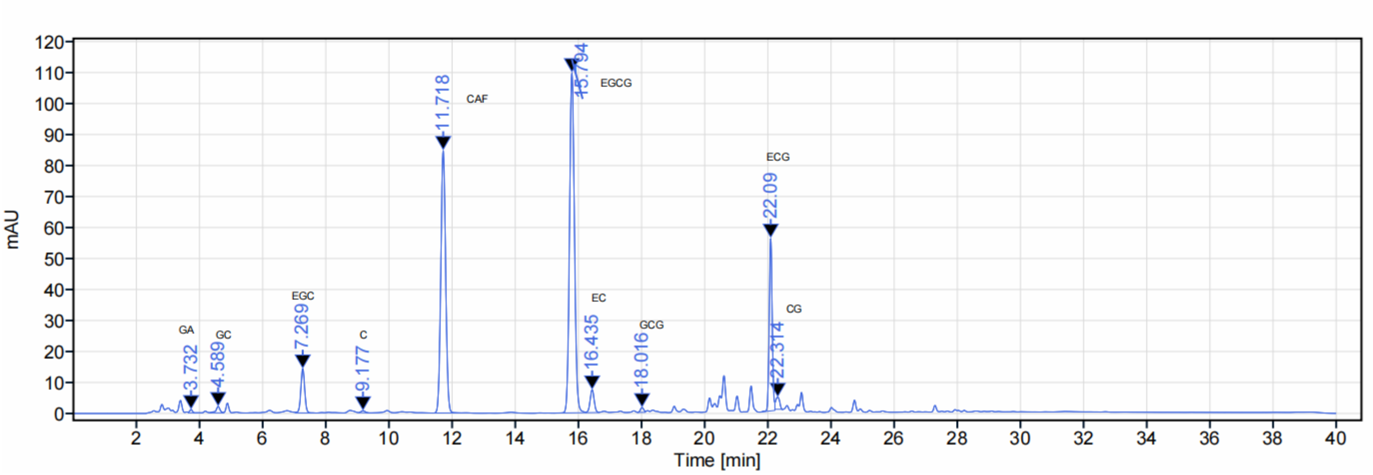

mAU
Time [min]
GA
GC
EGC
C
CAF
EGCG
EC
GCG
ECG
CG
3.732
4.589
7.269
9.177
11.718
15.794
16.435
18.016
22.09
22.314

(E) ZH

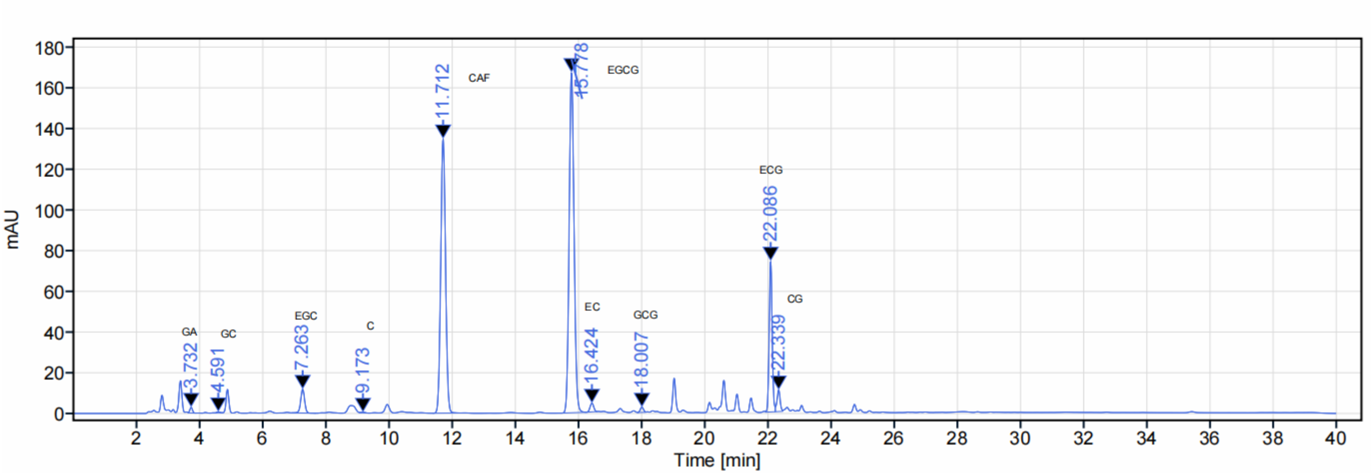
mAU
Time [min]
GA
GC
EGC
C
CAF
EGCG
EC
GCG
ECG
CG
3.732
4.591
7.263
9.173
11.712
15.778
16.424
18.007
22.086
22.339

(F) ZBH

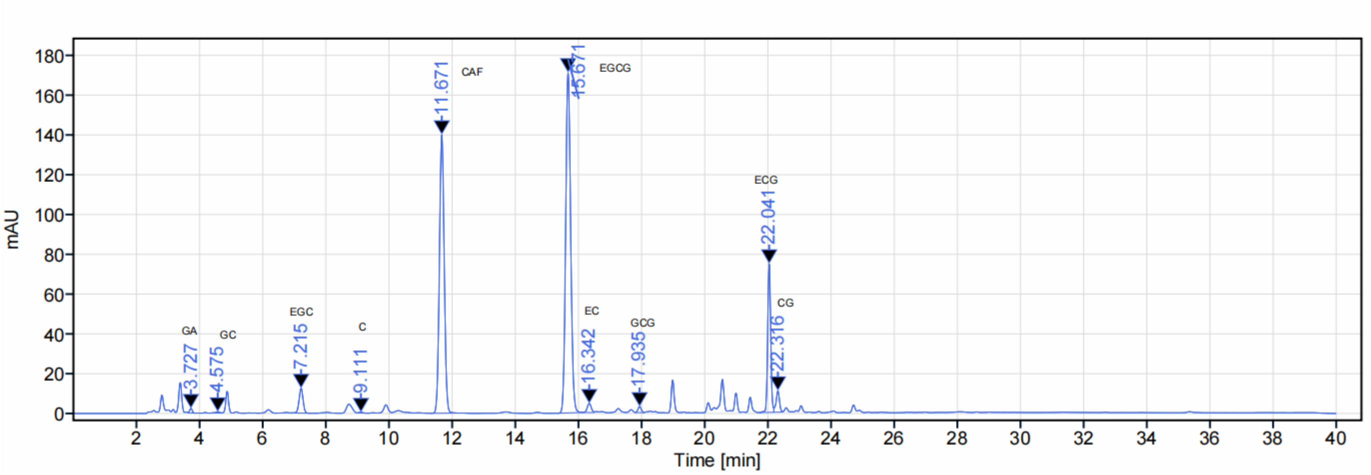

mAU
Time [min]
CAF
11.671
EGCG
15.671
ECG
22.041
GA
3.727
GC
4.575
EGC
7.215
C
9.111
EC
16.342
GCG
17.935
CG
22.316

(G) ZB

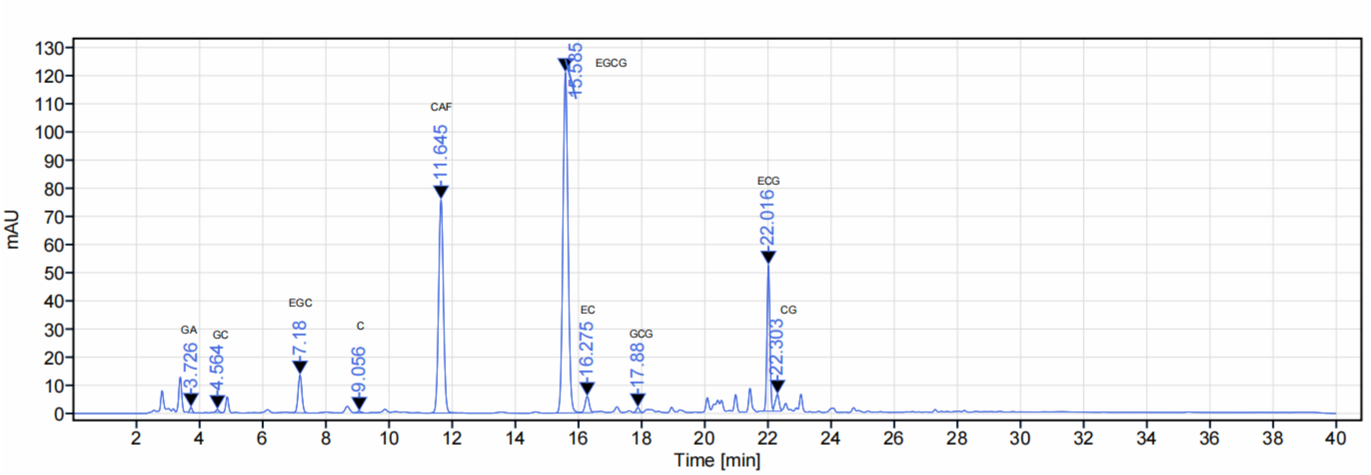
mAU
Time [min]
GA
GC
EGC
C
CAF
EGCG
EC
GCG
ECG
CG
3.726
4.564
7.18
9.056
11.645
15.585
16.275
17.88
22.016
22.303

(H) HSH

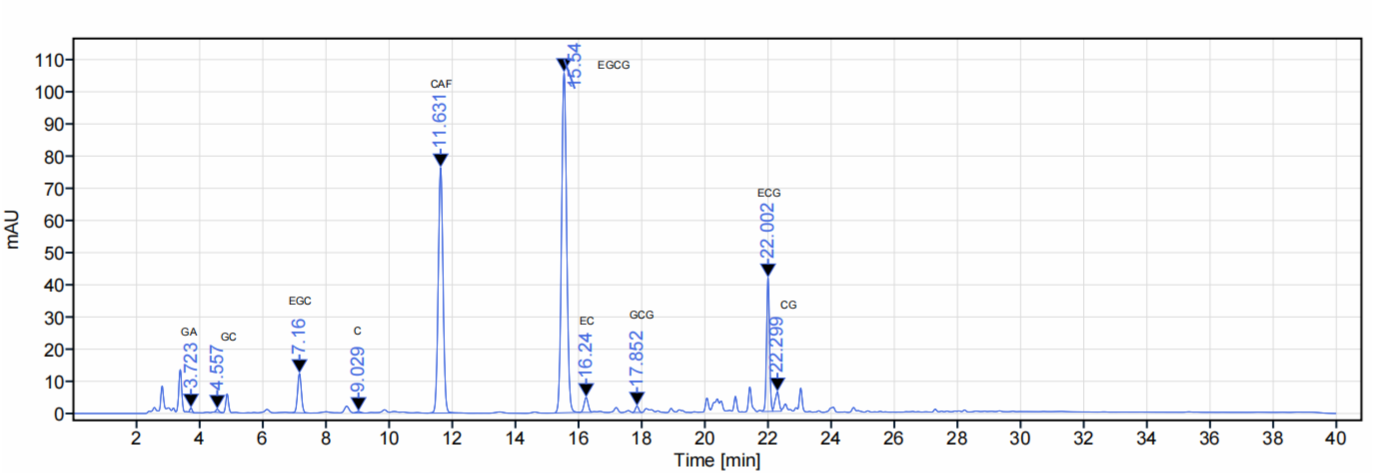
mAU
Time [min]
GA
GC
EGC
C
CAF
EGCG
EC
GCG
ECG
CG
3.723
4.557
7.16
9.029
11.631
15.54
16.24
17.852
22.002
22.299

(I) HS

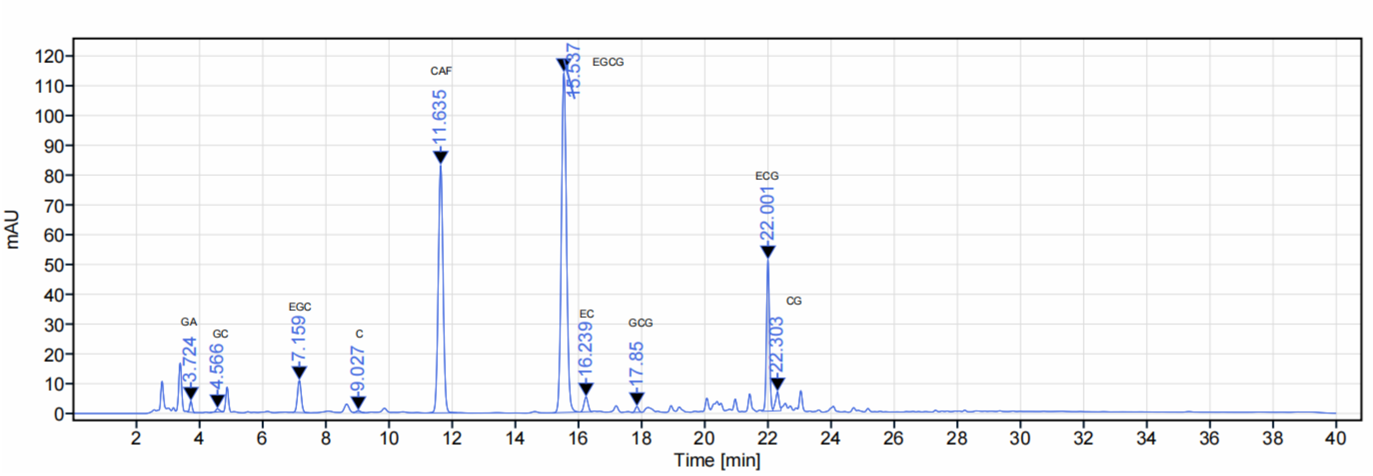
mAU
120
110
100
90
80
70
60
50
40
30
20
10
0
2
4
6
8
10
12
14
16
18
20
22
24
26
28
30
32
34
36
38
40
Time [min]
GA
3.724
GC
4.566
EGC
7.159
C
9.027
CAF
11.635
15.537
EGCG
EC
16.239
GCG
17.85
ECG
22.001
CG
22.303

(J) LJH

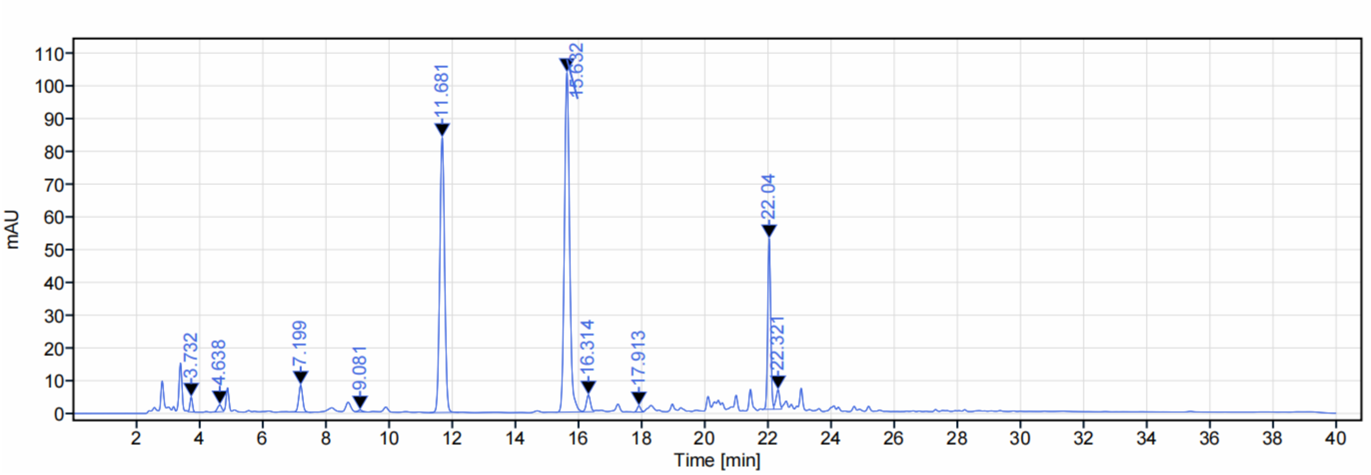

mAU
110
100
90
80
70
60
50
40
30
20
10
0
3.732
4.638
7.199
9.081
11.681
15.632
16.314
17.913
22.04
22.321
2
4
6
8
10
12
14
16
18
20
22
24
26
28
30
32
34
36
38
40
Time [min]

(K) LJ
