# Supplementary material for: Non-Volatile Metabolite Changes in Green Tea Following Helium-Flushed Enclosed-Chamber Pretreatment of Fresh Leaves Across Five Tea Cultivars
Source: Foods. 2026 Oct 21;15(20):3747. doi: 10.3390/foods15203747 (PMC13704259; doi:10.3390/foods15203747)
Supplement: Supplementary file 1 [file foods-15-03747-s001.zip › Supplementary File Description.pdf]

Supplementary File Description

The supplementary files in this folder are associated with the manuscript entitled "Non-Volatile Metabolite Changes in Green Tea Following Helium-Flushed Enclosed-Chamber Pretreatment of Fresh Leaves across Five Tea Cultivars," authored by Mi Hu, Yunqi Huang, Niuniu Shi, Wendi Zhang, Yihu Guan, Yapeng Li, Qianwen Sha, Qiongfen He, Qiaomei Wang, Xiujuan Deng, Wenxia Yuan, Run Yang, Huimin E, Xiaohui Wan, Mingshan Zhou, Baijuan Wang, and Xinghua Wang. The authors are affiliated with the College of Tea Science, Yunnan Agricultural University, Kunming 650201, China, and the College of Continuing Education, Yunnan Agricultural University, Kunming 650201, China.
